# Arrayable TDC with Voltage-Controlled Ring Oscillator for dToF Image Sensors

**DOI:** 10.3390/s25154589

**Published:** 2025-07-24

**Authors:** Liying Chen, Bangtian Li, Chuantong Cheng

**Affiliations:** 1 School of Electronics and Information Engineering, Tiangong University, Tianjin 300387, China; 2 Tianjin Key Laboratory of Photoelectric Detection Technology and System, Tianjin 300387, China; 3 Institute of Semiconductors, Chinese Academy of Sciences, Beijing 100083, China

**Keywords:** time-to-digital converter, TDC, LIDAR, linearity, dToF, sampling error

## Abstract

As the resolution and conversion speed of time-to-digital conversion (TDC) chips continue to improve, the bit error rate also increases, leading to a decrease in the linearity of TDC and seriously affecting measurement accuracy. This paper presents a high-linearity, low-power-consumption, and wide dynamic range TDC that was achieved based on the SMIC 180 nm BCD process. Compared with previous research methods, the proposed phase arbiter structure can eliminate sampling errors and improve the linearity of TDC. The preprocessing circuit can eliminate fixed errors caused by START and STOP signal transmission delays. Post-simulation results show that the TDC has high linearity, with ranges of DNL and INL being −0.98 LSB < DNL < 0.93 LSB and −0.88 LSB < INL < 0.95 LSB, respectively. The highest resolution is 156 ps, the maximum measurement time range is 1.2 μs, and the power consumption is 1.625 mW. The overall system architecture of TDC is very simple, and it can be applied to dToF LIDAR to measure photon flight time, capable of measuring a range of up to hundreds of meters, with an accuracy of 2.25 cm, high linearity, and without any post-processing or time calibration.

## 1. Introduction

TDC is an important component of systems based on Light Detection and Ranging (LIDAR) using the direct time-of-flight (dToF) principle [1], and an on-chip integrated TDC is usually used to accurately measure the time in flight of photons [2]. The LIDAR system emits photons and receives the returned photons through a photodetector, generating START and STOP signal pulses, respectively. Photon flight time is equal to the time interval between the START and STOP pulses, and the photon flight time is converted into a digital code by the TDC to indicate the distance to the target. At present, the dToF ranging method has become a popular feature development direction of 3D depth vision technology by virtue of its long ranging distance and better low-light performance, while the performance of TDC directly determines the ranging accuracy and error of dToF LIDAR [3,4,5,6].

Common types of TDCs are as follows [7]: (1) clock-period counter-type TDC, which uses a fixed-frequency clock to drive the counter counting principle, and the period T of this clock is the smallest unit of time quantization, with the advantage of a simple structure and a large measurement time range, and the disadvantage of a limited accuracy. (2) Delay line-type TDC: this adopts a series of CMOS gate delays in the delay line as the basic unit, with its minimum resolution depending on the gate delay time. Typically, in a 180 nm process, the transmission delay of an inverter is only about 50 ps. This structure overcomes the issue that the resolution index of traditional counter-type TDCs is restricted by the clock frequency. (3) Vernier TDC: its functional principle is analogous to that of a vernier caliper. It extends the gate-level delay line-based TDC to two delay lines, and its resolution is equal to the difference between the delay time of the fast delay line unit and that of the slow delay line unit, enabling a resolution lower than the gate delay. However, it is susceptible to process and environmental factors, which lead to variations in the delay time of each delay unit. Eventually, the difference between the two delay lines becomes larger, resulting in poorer linearity of the TDC [8]. (4) Counter-type + delay-lines type TDC: this hybrid structure of a multi-stage measurement TDC can improve the measurement accuracy while expanding the maximum measurement time range [9].

In summary, the combination of each single-mode TDC and segmented TDC constitutes a wide dynamic range and high-resolution TDC, and in the multi-segment TDC system architecture. The time to be measured in the lower segment TDC is actually the remaining time of the higher segment TDC, which is in essence the quantization of the time to be measured by a variety of TDCs with different resolutions. The key to realization is the articulation between the TDCs of each segment, that is, to satisfy the phase matching between adjacent segments [10]. Based on the foundation of multi-segment TDC realization, many novel structures have been born. For example, the ring oscillator type TDC, which has the advantages of a simple structure and robustness [11], is the most suitable type of TDC to be integrated into large-scale arrayed LiDAR chips.

In this paper, a high-performance gated ring oscillator type TDC for LIDAR applications is proposed. Based on gated edge triggering, a phase arbiter circuit is used to sample and hold the corresponding eight-phase rising edge positions in the VCRO at the moment of the onset of the START and STOP pulse signals, which effectively avoids the sampling error and improves the linearity of the TDC. Section 2 discusses the proposed TDC architecture and its operating principle. Section 3 describes the circuit implementation and design ideas. The simulation results are further analyzed in Section 4. Finally, Section 5 draws the conclusions.

## 2. Circuit Design Gated Ring Oscillator Type TDC and Principle of Operation

**Figure 1:**
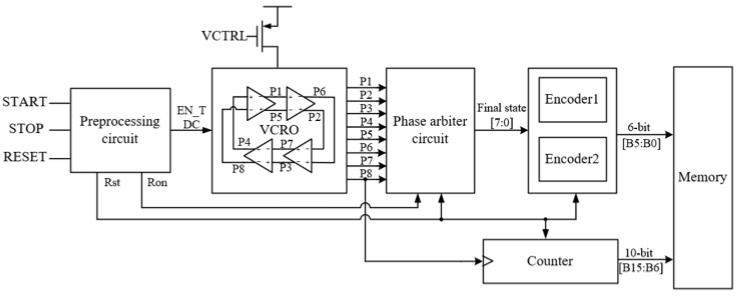
Structure of gated ring oscillator type TDC system.

The TDC, at the circuit level, uses a ring voltage-controlled oscillator-based TDC structure to quantify the time-of-flight, which is more compact and simpler compared to other types of TDCs (for example vernier type TDCs) [9,12]. The block diagram of the gated ring oscillator type TDC module proposed in this paper is shown in Figure 1. The system mainly consists of a voltage-controlled ring oscillator (VCRO) circuit, preprocessing circuit, counter, encoder, phase arbiter circuit, and memory.

The core of the TDC is a four-stage pseudo-differential voltage-controlled ring oscillator with VCRO as shown in Figure 1, which is controlled by a PLL generating a stable VCTRL voltage to obtain the desired oscillation frequency [13], thus expanding the frequency tuning range and improving the overall TDC measurement performance. Firstly, the START signal, STOP signal, and RESET signal go through a preprocessing circuit to generate the enable signals Ron, Rst, and EN_TDC to control the other modules to work. The fine resolution of the TDC is determined by the eight uniform phases (P1–P8) generated by the VCRO, and the minimum resolution is equal to the magnitude of the time of one phase difference, defined as P; the sampling circuits are used to control the frequency of the TDC at the moment when the START and the preprocessing circuit samples and holds the eight split-phase clocks at the onset of the START and STOP signals, respectively, to form an 8-bit binary code with phase position information, which is encoded by the encoder and converted into two 3-bit phase signals. The P8 phase output is followed by a 10-bit asynchronous counter for coarse counting, and the size of the coarse counting time for a cycle is equal to 8*P. The overall system architecture of the TDC is very simple and does not require the use of an FPGA or processor to perform the time-to-digital conversion.

**Figure 2:**
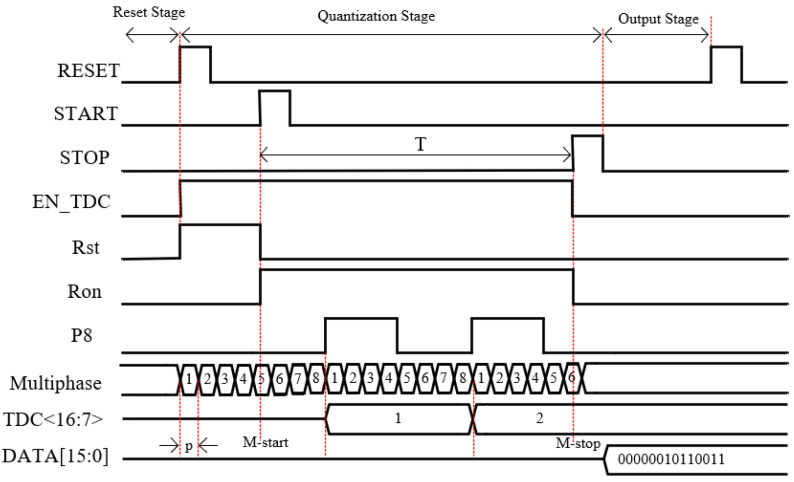
Timing schematic of the TDC quantization process.

In order to achieve smaller resolution, an accurate sampling of the START and STOP signals at the moment of their onset is required to correspond to partial-phase clock-phase information within the VCRO [10]. Conventional sample-and-hold circuits consist of switching devices, capacitors, and operational amplifiers. The response speed and accuracy of this circuit structure are not suitable for phase sampling of TDC [14]. The sampling circuit proposed in this paper is described in detail in Section 3. Figure 2 illustrates the timing principle of the TDC quantization process in this design. START and STOP pulse signals arrive at the time that the sampling circuit samples the eight split-phase clock phases to generate an 8-bit binary code consisting of 0 or 1. The encoder then converts this special 8-bit binary code into a 3-bit code that represents the fine resolution results. The counter is incremented by one when the VCRO cycles through eight phase changes.

The time interval expression to be measured is as follows:

(1) $$T=\left(N\times 8-M_{Start}+M_{Stop}\right)\times P$$

where N is the counter reading and is equal to the encoder 1 and encoder 2 output code corresponding to the decimal value and P product.

## 3. TDC Circuit Design and Implementation

**Figure 3:**
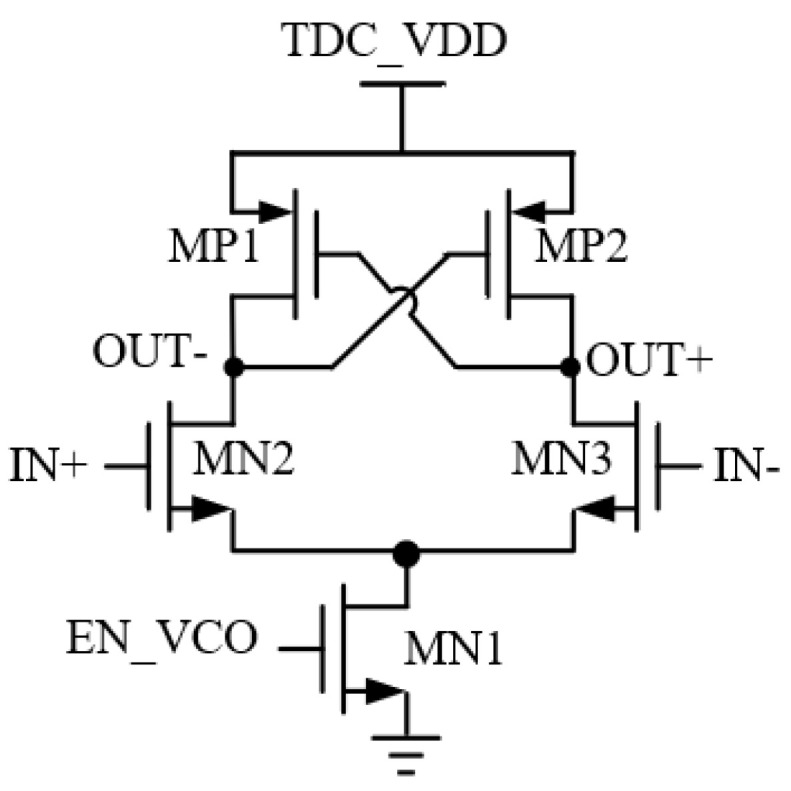
Pseudo-differential delay unit.

The voltage-controlled oscillator in this article adopts a ring oscillator design [15]. In order to meet the requirements of TDC single-pixel integration, key parameters such as area, power consumption, and time accuracy need to be synergistically optimized. At the same time, a four-stage ring oscillator is used to suppress common mode interference, and the circuit structure is shown in Figure 3.

Compared to the differential delay unit voltage-controlled oscillator (VCO), which has a wide tuning range (>50%) and high frequency capability (GHz level) suitable for reconfigurable RF systems, the fully integrated relaxation oscillator exhibits disruptive advantages in the mid-low-frequency high-precision clock field: the differential VCO has a temperature drift > 500 ppm/°C due to the temperature sensitivity of the delay chain, while the relaxation architecture compresses the temperature drift to 23.4 ppm/°C through voltage delay feedback (VDF), optimizing the voltage sensitivity to ±0.15%/V [16]. In terms of noise performance, the relaxation oscillator achieves 156 dBc/Hz FOM with switch capacitor swing enhancement (SCSB) and self-threshold calibration (CSTA), significantly better than the typical −140 dBc/Hz FOM of the differential VCO at −150 dBc/Hz (limited by circular structure flicker noise). In terms of power consumption, the relaxation scheme only consumes 51.4 μW at 1.6 MHz, which has a numerical advantage over the GHz level differential VCO (>100 μW) [17]. The essential trade-off lies in the following application scenario: the differential VCO is suitable for wideband systems such as software defined radio, while the relaxation oscillator, with its ultra-stable frequency and low phase noise at the near end, has become the optimal clock solution for biosensing and IoT nodes.

In the design of ring oscillator architecture, the four-stage topology exhibits unique stability and gain optimization mechanisms compared to the three-level implementation: the four-stage ring oscillator introduces a first-stage non-reverse buffer unit to maintain the equivalent oscillation phase of 360° while breaking the potential DC balance point locking risk of symmetrical topology [18]. This structure satisfies the Barkhausen criterion, and the oscillator oscillation condition is shown in Equation (2):

(2) $$H_{delay}\left(s\right)=\frac{-A}{1+\frac{s}{\omega }}$$

In the formula, *ω* is the pole of the unipolar delay module, and *A* is the circuit gain. The overall loop gain can be obtained as shown in Equation (3):

(3) $$H_{ALL}\left(s\right)=-\frac{A^{4}}{{\left(1+\frac{s}{\omega }\right)}^{4}}$$

In the analysis of the starting frequency of a ring oscillator, based on the Barkhausen stability criterion, the total phase shift of the four-stage cascade is 180°, which satisfies the oscillation phase condition, with a single-stage phase shift of 45°. The oscillation frequency at this time can be obtained through Formula (4), and by substituting *ω_osc_* = *ω*, the circuit gain can be obtained as shown in Formula (5):

(4) $$arctan\left(\frac{\omega_{osc}}{\omega }\right)=45\;{}^{\circ}C$$

(5) $$A=\sqrt{1+{\left(\frac{\omega_{osc}}{\omega }\right)}^{2}}=\sqrt{2}$$

**Figure 4:**
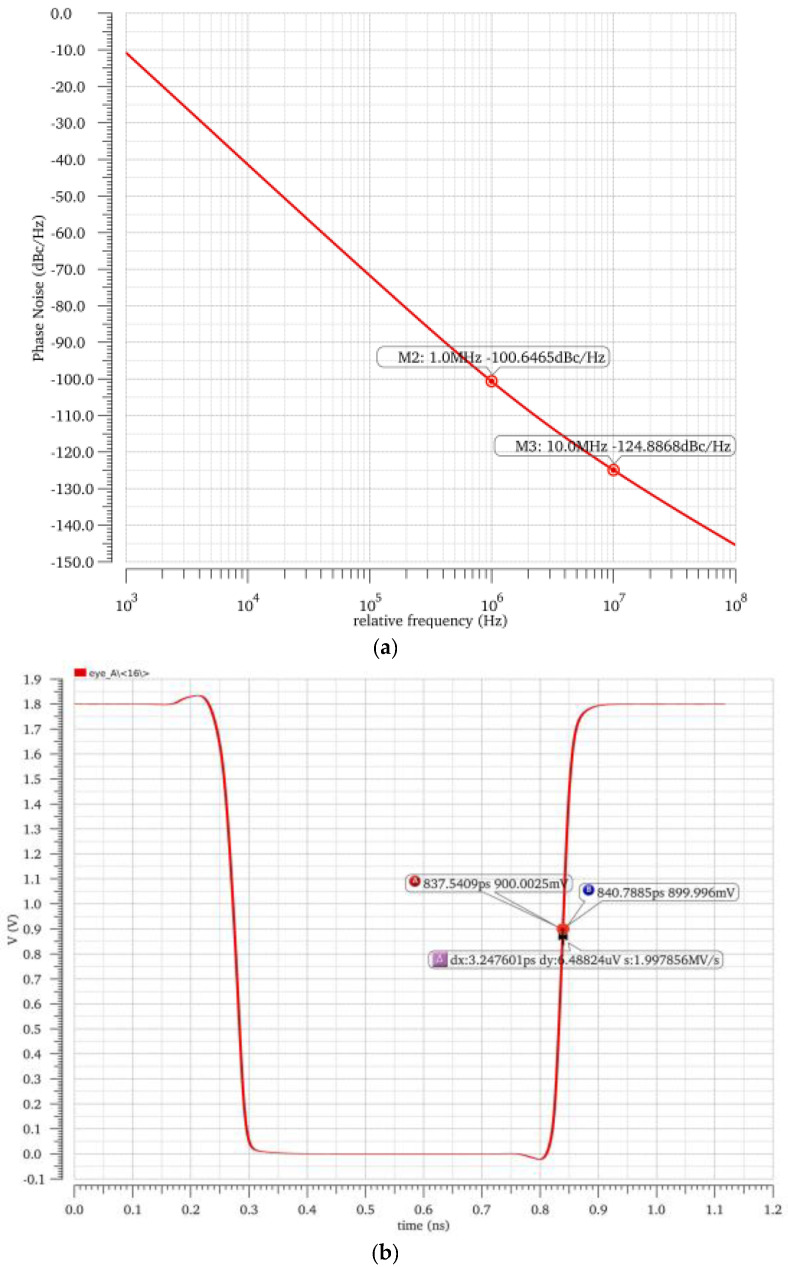
Simulation results of phase noise (**a**) and split-phase clock jitter of VCRO (**b**).

The delay circuit of the ring oscillator adopts a pseudo-differential pair delay unit circuit, as shown in Figure 3. The positive-feedback quasi-differential inverter unit circuit consists of a positive feedback section and a differential inverter circuit, compared to the cascaded NMOS transistor MN1 used as the main control switch in this paper. The function of MN1 is to control the on and off of the ring oscillator. EN_VCO is the pulse-level signal in the preprocessing circuit. The MP1 tube and MP2 tube cross-coupling architecture and differential pair input synergy accelerate the flipping of output nodes. During the effective high-level phase of EN_VCO, the MN1 transistor conducts, and when the IN + voltage at the input gate terminal of the MN2 transistor is high, it conducts deeply. The OUT-voltage is quickly pulled down, and at this time, the gate voltage of the MP2 transistor synchronously decreases. The MP2 transistor opens and pulls up the OUT + node voltage to a high level. When the IN-voltage is low, the increase in OUT + voltage causes the MP1 transistor to turn off. At this time, it competes with the MN2 transistor and MP1 transistor for current at the OUT-node, accelerating the OUT-voltage to eventually stabilize at a low level. Using MN1 tubes to form a tail current source to control the charge and discharge current path can effectively suppress the effects of non-ideal factors such as process angle deviation, device misalignment, temperature offset, noise interference, and substrate coupling effects on the circuit. It can significantly improve the circuit’s ability to suppress common-mode noise in power supply, and its power supply suppression bit performance is significantly improved compared to traditional structures. Specter simulation results are shown in Figure 4. The phase noise is −100.64 dBc/Hz and −124.88 dBc/Hz at 1 MHz and 10 MHz, respectively, and the split-phase clock jitter is at 3.24 ps.

**Figure 5:**
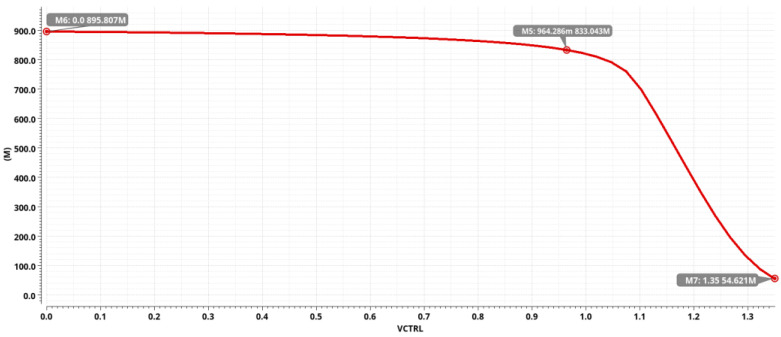
$K_{VCO}$ gain curve graph.

The VCTRL voltage provided by the PLL controls the oscillation frequency of the VCRO and thus the resolution of the TDC [19]. The variation in VCTRL voltage concerning the oscillation frequency of VCRO is scanned by simulation after the tt process angle at 25 °C. The simulation results are shown in Figure 5; according to the changing relationship between the oscillation frequency and resolution of VCRO and VCTRL voltage, the average gain of VCRO during the changing of VCTRL voltage in 0 V–1.35 V is 622 MHz/V. To obtain the VCTRL control voltage, it is necessary to provide a voltage after locking the frequency of the PLL; the PLL’s oscillator structure and the TDC’s VCRO keeping in line can improve the linearity of the TDC’s VCRO by more than 99% linearity [20]. This control voltage given by the PLL can effectively compensate for the effect of PVT variations on the TDC resolution. By adjusting the VCTRL voltage (0 V–1.35 V), the corresponding resolution variation can be achieved in the range of 139 ps–2.3 ns.

With the continuous improvement of TDC time resolution and sampling clock frequency, the problem of sampling metastability occurs, and the bit error rate also increases, seriously affecting the accuracy of TDC [10,21]. This phenomenon is due to the random arrival of photons, and the generated STOP signal pulse is also uncertain. When the rising edge of the input signal CK does not meet the sampling conditions, it is impossible to accurately determine whether photons have arrived.

**Figure 6:**
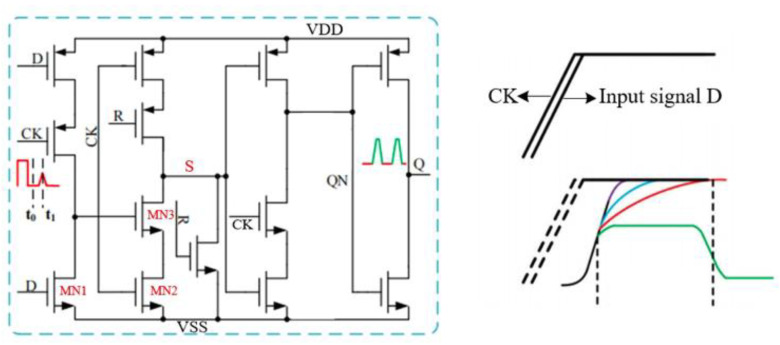
D flip-flop metastable state.

The transistor level structure of the D-type flip-flop using TSPC dynamic logic structure is shown in Figure 6.

When the edge of the input signal and the edge of the sampling clock satisfy the timing competition condition, a transistor-level conflict will occur inside the D flip-flop, causing MN3 located on the same pull-down path to turn off and MN2 to turn on. Therefore, the control line S is at an uncertain intermediate potential, causing the output of the final D flip-flop to be unable to stabilize accordingly. The probability of metastable output occurrence is closely related to the process used by the trigger and the rising speed of the input edge. Usually, there is a strong positive correlation between the probability of metastable failure and the integrity of process nodes and signals. As the process size increases, the saturation current decreases, the input edge becomes gentler, and the transition time becomes longer, resulting in a higher probability of metastable state occurrence.

**Figure 7:**
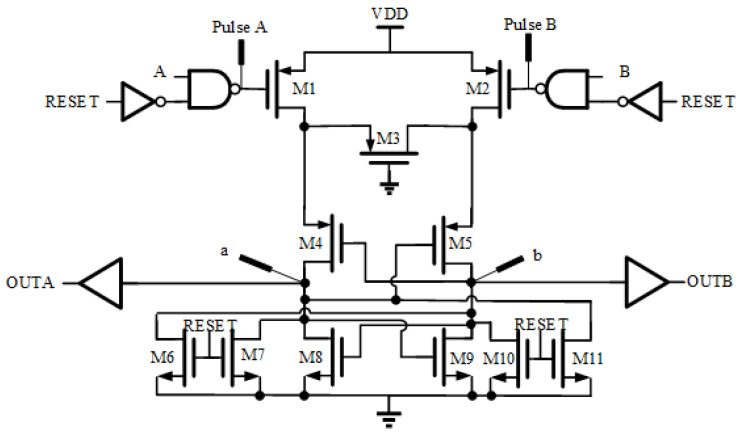
Schematic diagram of phase arbiter.

The resolution of TDC is determined by the high-frequency phase separated clock frequency and the sample and hold circuit. This paper proposes a phase arbiter circuit structure for the sample and hold circuit of TDC, as shown in Figure 7: the circuit consists of a comparator, a reset circuit, and an edge detector. This circuit has a completely symmetrical topology structure with good matching, which can reduce the sensitivity of the sample and hold the circuit to external environmental changes.

When TDC quantizes the time interval between START and STOP pulse signals, the rising edge is used as a reference, so the phase arbiter adopts rising-edge detection. The working principle of the circuit is that the A and B ports on the left and right sides are used to input the phase signal to be detected; RESET is effective at high levels, while the phase arbiter works at low levels. At high levels, it not only resets but also turns off the input of the signal to be detected, effectively reducing the overall power consumption of the circuit. Adding an inverter chain as an output buffer between nodes a and b and output ports OUTA and OUTB can reduce the impact of the load circuit and improve the ability to drive the next level load.

**Figure 8:**
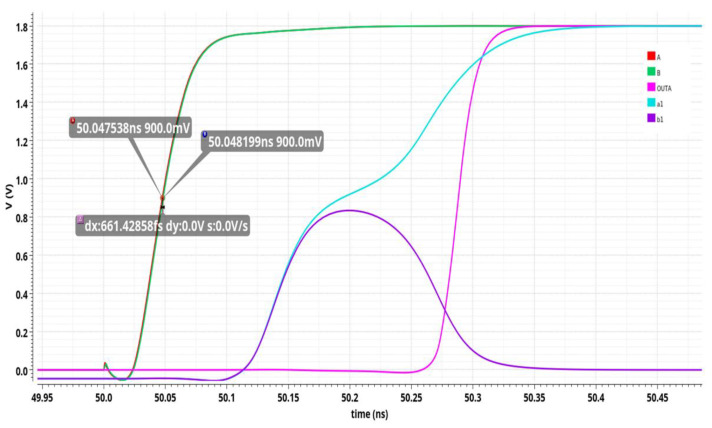
Simulation results of phase arbiter.

The transient simulation results of the phase arbiter are shown in Figure 8. Provide the minimum interval between pulse input signals A and B. When signal A leads signal B, it can be seen that node PulseA is pulled down earlier than the PulseB signal. At the same time, the levels of nodes a and b are also raised, but the PulseA potential is pulled down earlier, causing the M1 transistor to turn on first. Therefore, the speed at which the potential at point a is raised is faster than that at point b. When the voltage of node a exceeds the threshold voltage of the MOS transistor, M5 transistor is turned off, M9 is turned on, and the level of node b is pulled to ground, completing a phase comparison. The phase arbitration result this time is that the OUTA port outputs a high level, the OUTB port outputs a low level, and vice versa; this also holds for the B signal leading the A signal.

In the pixel built-in clock scheme, each pixel contains a VCRO clock source. If the VCRO in the pixel is constantly oscillating, it will inevitably generate significant power consumption [10]. To reduce power consumption, it is necessary to control the working state of the VCRO in the pixel; if the pixel TDC has a quantization requirement, the VCRO oscillation is built-in, otherwise the pixel VCRO should be in standby mode.

**Figure 9:**
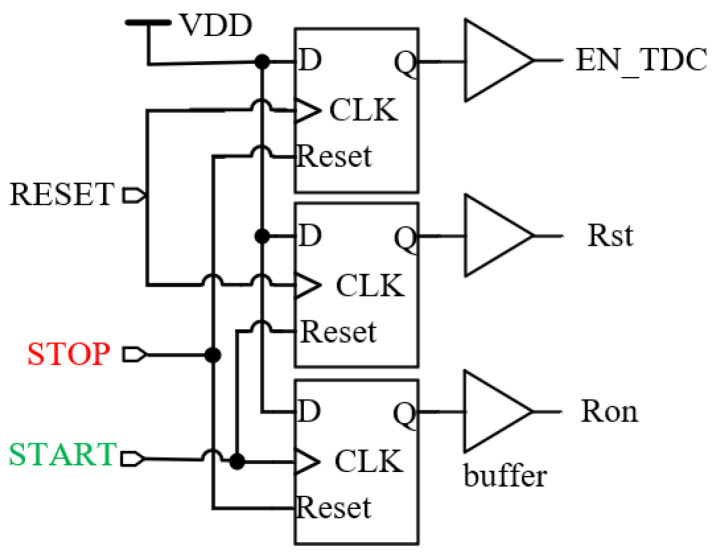
Preprocessing circuit structure.

In order to effectively save power consumption, a preprocessing circuit is proposed to control the start/stop of the next level module. The circuit structure is simple, as shown in Figure 9. The preprocessing circuit consists of three D flip-flops with a reset function and buffer. Its function is to preprocess the START signal and STOP signal, generate rectangular pulse signals with edge information, and use them for starting/stopping other sub-modules of TDC. This cross structure can reduce the difference in propagation delay between START and STOP signals in the circuit, improving the accuracy of TDC measurement without the need for subsequent time correction processing.

**Figure 10:**
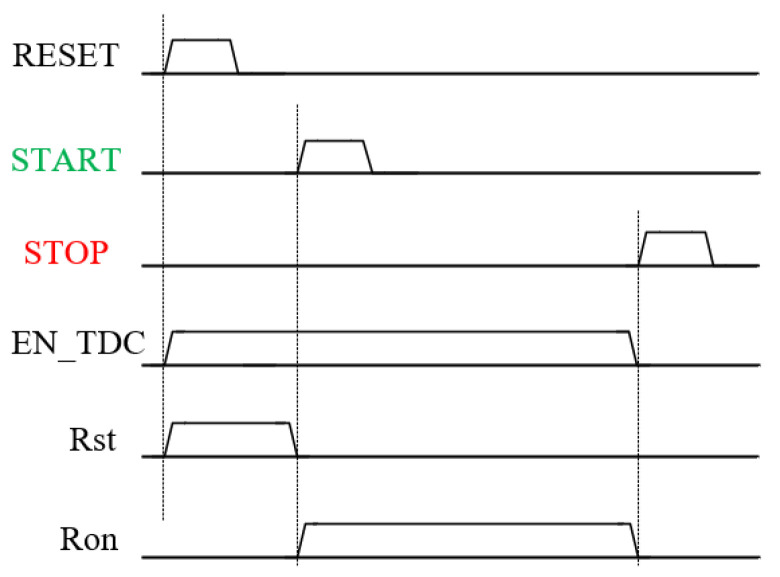
Timing diagram of preprocessing circuit.

The working timing diagram of the preprocessing circuit is shown in Figure 10. When the rising edge of the RESET signal arrives, the EN_TDC and Rst signals become high-level. When the EN_TDC high-level is reached, the MN1 transistor of the VCRO is turned on, and the power to ground forms a path for the oscillator to start oscillating and quickly stabilize at the PLL locked frequency. When Rst is high, it resets the counter, encoder, and sample and hold circuit. At this time, all three modules are in a closed state, and the power consumption of the circuit is very low. When the rising edge of the START signal arrives, the Ron signal becomes high and the Rst signal becomes low, and the counter and encoder are in operation at this time.

## 4. Analysis and Discussion of Simulation Results

**Figure 11:**
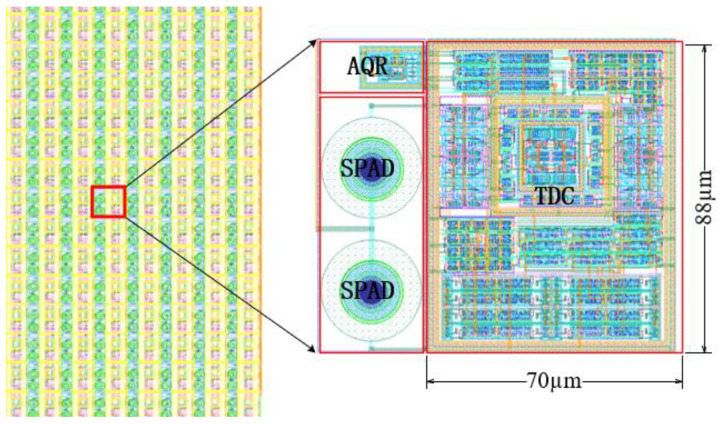
TDC layout.

The TDC chip design is fabricated using the standard SMIC 180 nm BCD CMOS process, the overall area is shown in Figure 11, the TDC layout area is 70 μm × 88 μm, and there are mainly preprocessing circuits, VCRO, encoders, counter, and a memory circuit, which do not include the peripheral circuits connected to the pin pad. At a supply voltage of 1.8 V and 25 degrees Celsius, the post-layout simulation results show an average current of 903.1 μA and a power consumption of 1.625 mW.

**Figure 12:**
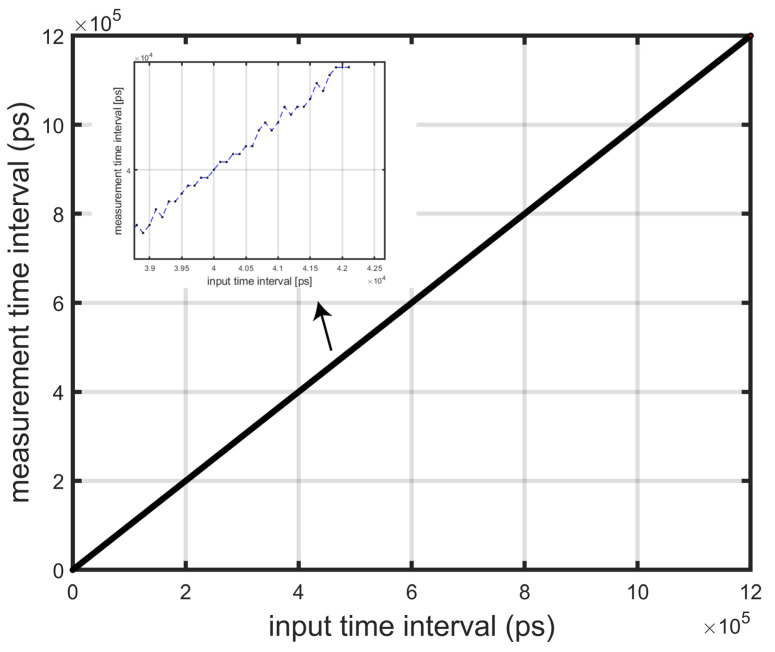
TDC post-layout simulation.

The test of static characteristics of TDC circuits includes dynamic range, time resolution, and nonlinear error [7]. The dynamic range and time resolution can be expressed by constructing the transfer characteristic curve, and the nonlinear error can be obtained according to the transfer characteristic curve. The specific test method of the transfer characteristic curve is as follows: firstly, the time interval between START and STOP is stepped at an interval of 500 ps of the coarse counting period, which is used to detect the dynamic range of the circuit measurement. Then a few critical times are selected as START points and stepped in the same way with a time length of 10 ps, and then the input time intervals corresponding to the jumps in the output results are recorded, and the time intervals between the two jump points are the actual time resolution. The statistical input time interval STARTs from 10 ps to 1.2 μs, and the Monte Carlo simulation transfer curve of the TDC is shown in Figure 12. The measured input time interval and the measurement time interval can maintain a strong linear relationship, in which the nonlinear error is less than one resolution of 156 ps.

**Figure 13:**
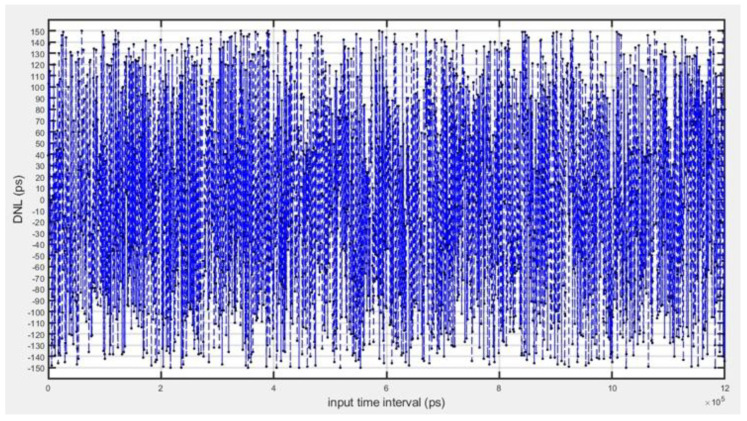
TDC-measured nonlinear error DNL.

**Figure 14:**
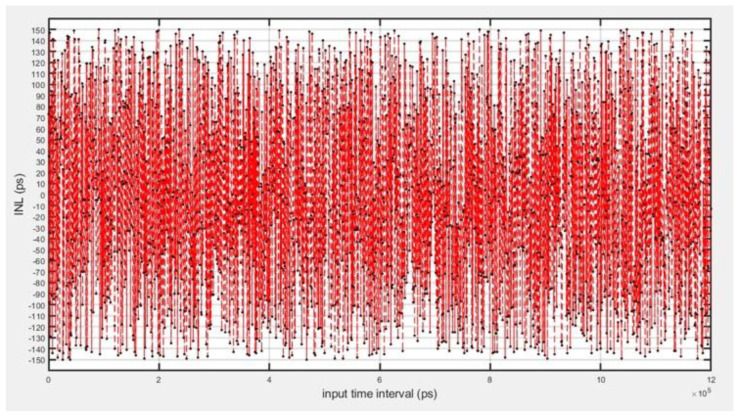
TDC-measured nonlinear error INL.

In evaluating the nonlinearity error of the TDC system, differential nonlinearity (DNL) is shown in Figure 13, and integral nonlinearity (INL) curves were obtained as shown in Figure 14, based on all the data obtained from the 0 to 1.2 s resolution test. The results show that −0.98 LSB < DNL < 0.93 LSB and −0.88 LSB < INL < 0.9V5 LSB, which are both less than ±1 LSB (1 LSB = 156 ps), indicating that the TDC circuits have a high degree of linearity and a small range of error fluctuations.

Table 1 summarizes the key performance metrics of the proposed TDC in comparison with existing techniques. A paper [1] presented in 2021 uses address latches to store the signals at the moment when STOP comes, which enables a single TDC to measure multiple events. Ref. [10] does this by combining a Union Reference Counter (URFC) with an interpolated delay line to obtain high accuracy and a wide dynamic range. However, its sampling circuit uses a D flip-flop, which has insufficient sensitivity and is prone to introducing nonlinear errors during high-frequency clock-phase sampling. One should adopt a comparator structure circuit to do the sampling, which effectively reduces the occurrence of sampling error and improves the linearity of the TDC, which also requires time correction processing, has a more complex structure, and the dual delay loop is susceptible to PVT. Ref. [22] proposed a gated ring oscillator (GRO) architecture to reduce the area and power consumption. Nevertheless, it adopted a D flip-flop for the sampling with a resolution of 1.76 ns at 1 KS/s sampling frequency. There is also a paper [23] which used a D flip-flop to do the sampling; limited by the D-trigger, the minimum resolution was only 36.2 ps. With the phase arbiter circuit proposed in this paper, the resolution can be further improved. Compared with previous work, the TDC circuit designed in this paper achieves a large dynamic range with high linearity while maintaining a relatively small-time resolution. Meanwhile, other performance parameters of the TDC such as area and power consumption are controlled within reasonable limits [24].

## 5. Conclusions

In this paper, a new phase arbiter and preprocessing circuit technique is proposed and applied to a TDC design, which can achieve high linearity, low power consumption, and no subsequent time correction. The TDC chip is designed using the SMIC 180 nm BCD process, and post-layout simulation results verify the effectiveness of the technique. The simulation results show an average current of 903.1 μA at a supply voltage of 1.8 V, a power consumption of 1.625 mW, a minimum resolution of 156 ps, maximum measurement time range of 1.2 μs, and high linearity. This TDC for dToF LIDAR photon time-of-flight measurement has a simple architecture and can measure distances as low as 2.25 cm and as high as thousands of kilometers, making it ideal for use in applications such as rangefinders, dToF LIDARs, UAVs, and robotic advanced pilot assistance systems.

## Figures and Tables

**Table 1:** Key performance comparisons of the TDC.

Parameter	This Work	[24]	[1]	[20]	[23]	[2]
Technology Node/nm	180	180	65	130	40	150
Architecture	VCRO	VCRO	DLL	GRO	Branching	GRO
Max range (μs)	1.2	-	0.64	1.76	0.148	0.053
Fill factor	11.15%	70%	49.7%	-	-	19.48%
Resolution/ps	156	208	97.65	1760	36.2	210.2
DNL/LSB	−0.98~0.93	−0.52~0	−0.4~0.6	-	1.3	1.28
INL/LSB	−0.88~0.95	−0.49~0.73	−0.4~0.7	-	3.61	1.92
Power(W)	1.625 m	-	-	45.85 n	0.28 m	-
TDC Area(μm^2^)	6160	31,000	-	-	43,000	402.7

## Data Availability

The original contributions presented in this study are included in the article. Further inquiries can be directed to the corresponding author.
